# Supplementary material for: Anti-Inflammatory Effect of Izalpinin Derived from Chromolaena leivensis: λ-Carrageenan-Induced Paw Edema and In Silico Model
Source: Molecules. 2023 Apr 26;28(9):3722. doi: 10.3390/molecules28093722 (PMC10179959; doi:10.3390/molecules28093722)
Supplement: Supplementary file 1 [file molecules-28-03722-s001.zip › molecules-2347014-supplementary.pdf]

Supplementary Material

Anti-inflammatory effect of izalpinin derived from *Chromolaena leivensis*: λ-carrageenan-induced paw edema and in silico model

Juan C. Mancipe[1], Pedro Vargas-Pinto[1], Oscar E. Rodríguez[2,3], Paola Borrego-Muñoz[2], Iovanna C. Castellanos Londoño[1], David Ramírez[4], Luis G. Piñeros[2], María Camila Mejia[2] and Luis M. Pombo[2]*

*Corresponding author: Luis M. Pombo

E-mail address: miguel.pombo@juanncorpas.edu.co

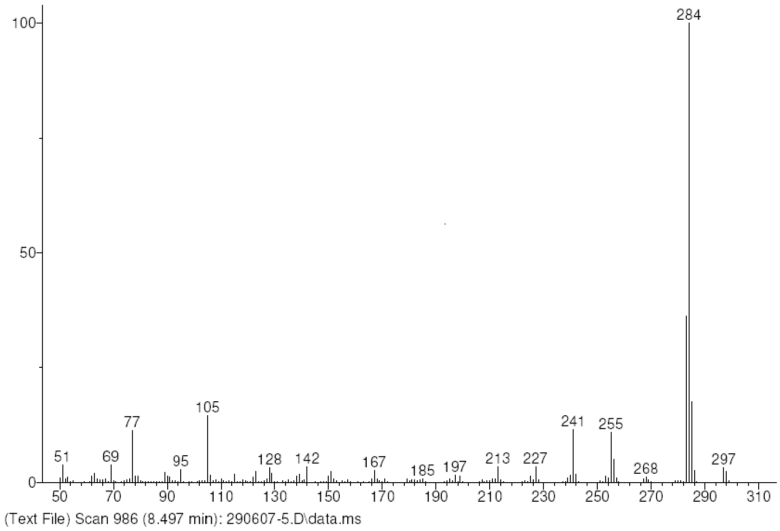

Figure S1. Mass spectra of izalpinin

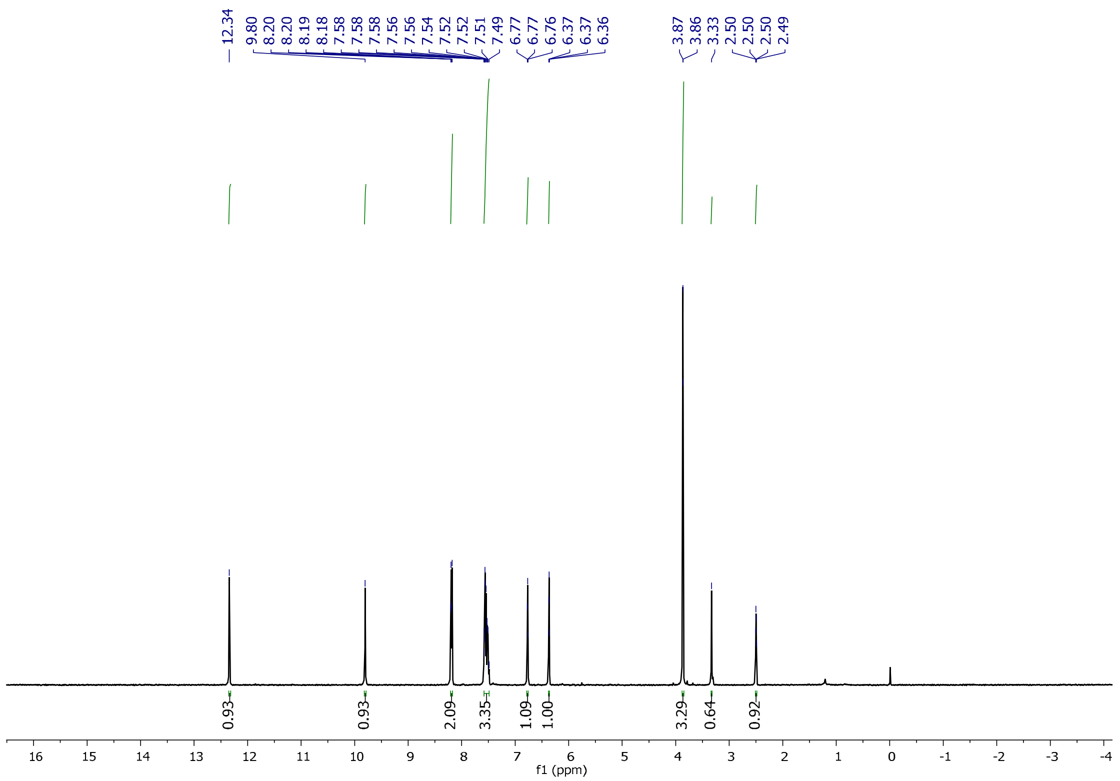

Figure S2. ^{1}H spectra of izalpinin.

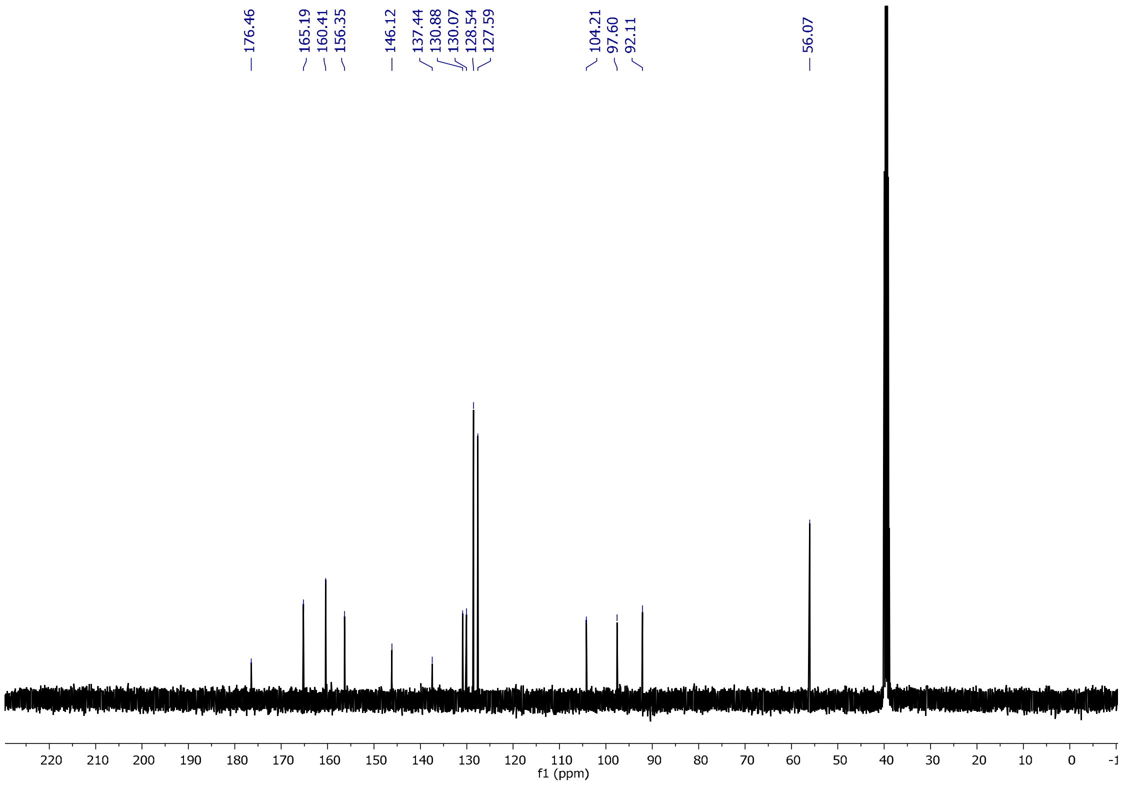

Figure S3. ^{13}C spectra of izalpinin.

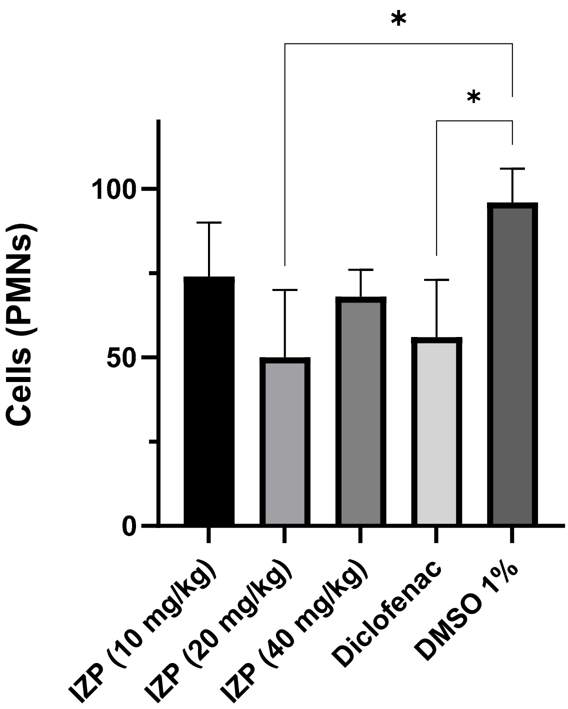

Figure S4. Polymorphonuclear leukocyte count in the histopathological slides obtained from the experimental groups. Cell counting was performed using a Zeiss optical light microscope. The PMNs present in five fields were counted using a 100x objective. Only differences (lower number of PMNs) are observed in the group treated with IZP at 20 mg/kg.

*$p<0.05$ (one-way ANOVA, Dunn's multiple comparisons test) with respect to the negative control (DMSO 1%).

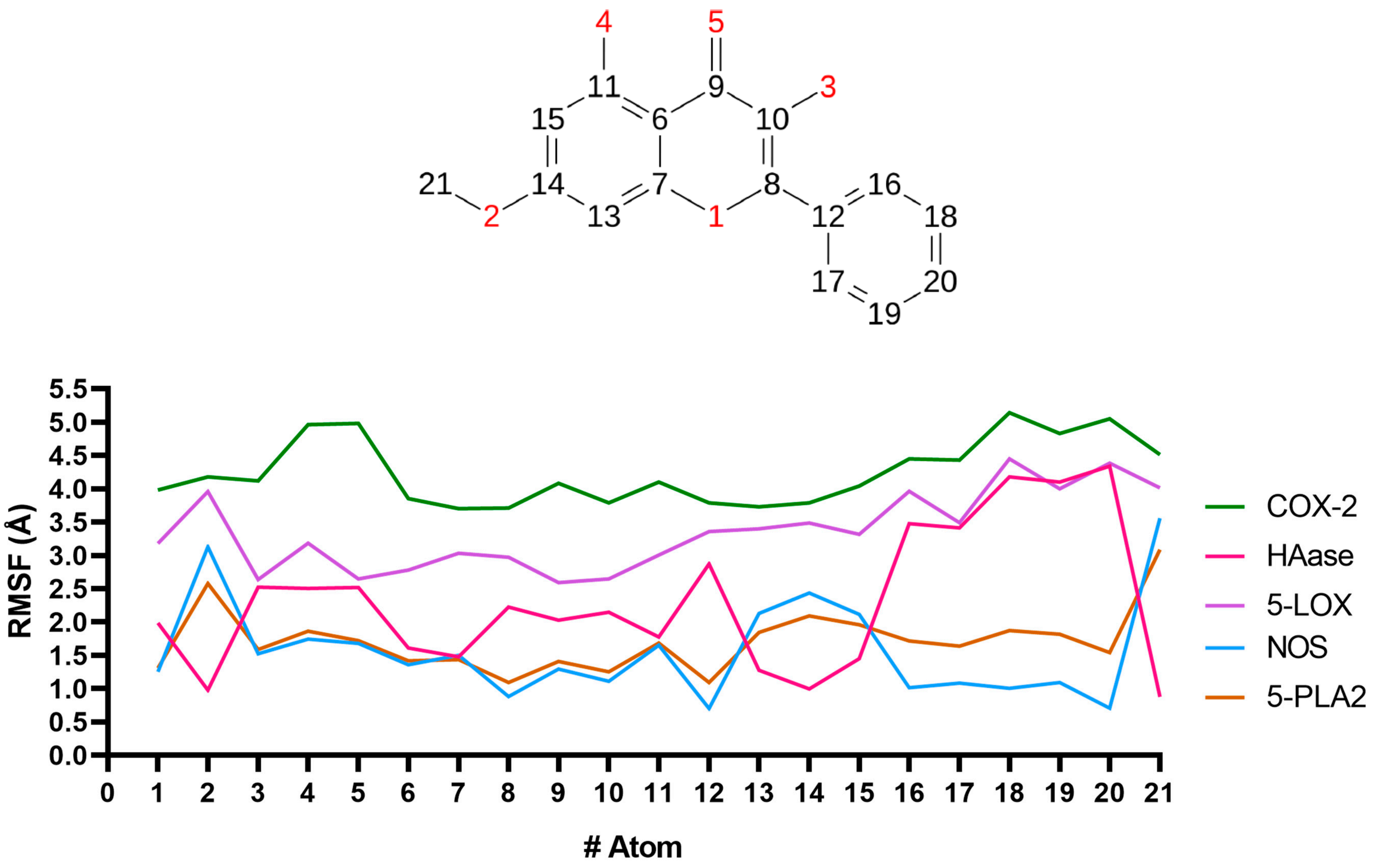

Figure S5. Root Mean Square Fluctuation (RMSF) of IZP.

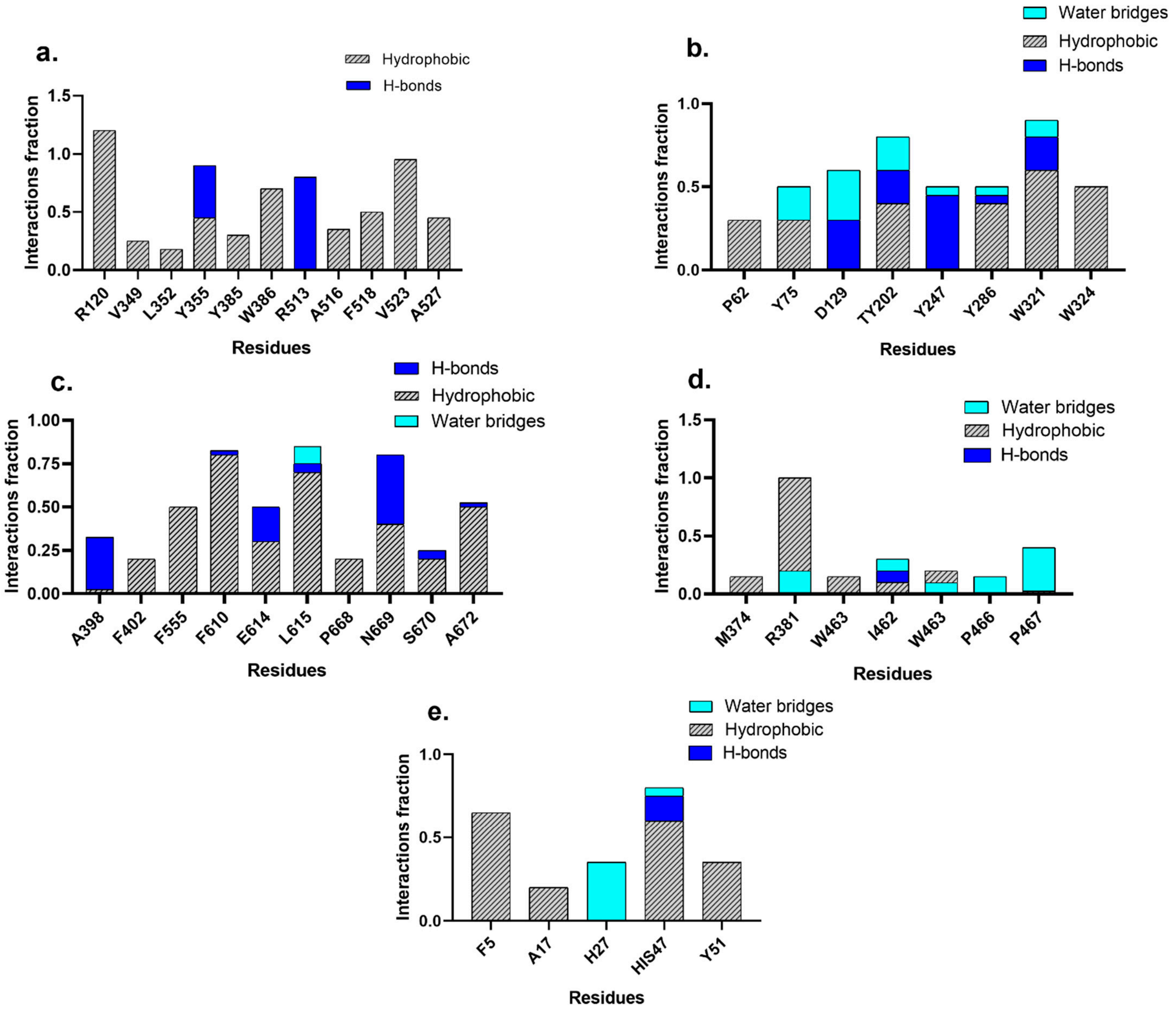

Figure S6. Stacked bar chart representation of IZP with active site amino acid residues of receptors; a. COX-2; b. HAase; c. 5-LOX; d. NOS; e. 5-PLA2.

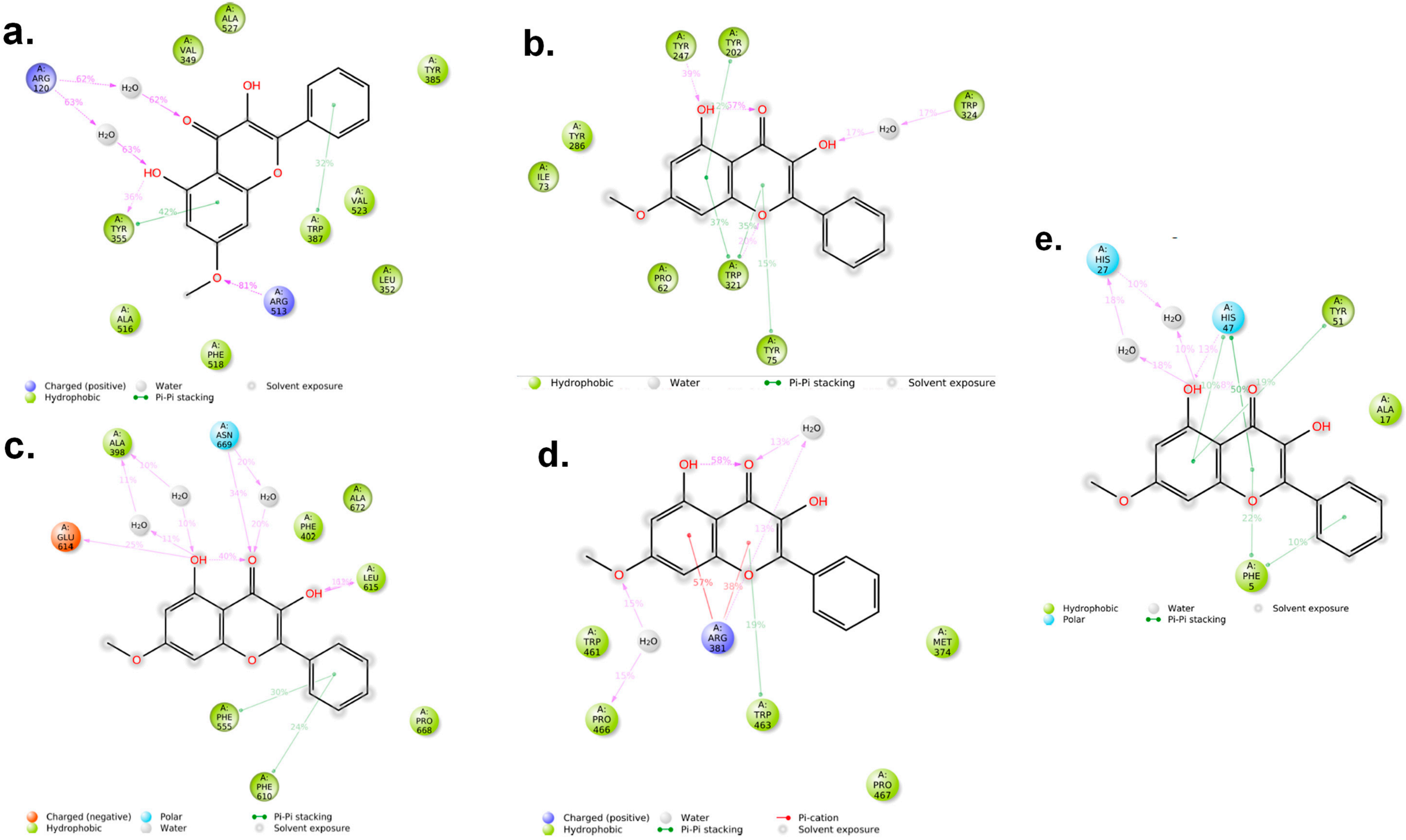

Figure S7. Schematic of detailed IZP atom interactions with the protein residues; a. COX-2; b. HAase; c. 5-LOX; d. NOS; e. 5-PLA2.
